# Wogonin Has Multiple Anti-Cancer Effects by Regulating c-Myc/SKP2/Fbw7α and HDAC1/HDAC2 Pathways and Inducing Apoptosis in Human Lung Adenocarcinoma Cell Line A549

**DOI:** 10.1371/journal.pone.0079201

**Published:** 2013-11-12

**Authors:** Xin-mei Chen, Yang Bai, Yu-jian Zhong, Xiao-lin Xie, Han-wu Long, Yu-yin Yang, Shi-gen Wu, Qiang Jia, Xiao-hua Wang

**Affiliations:** 1 Guangzhou Medical University, Guangzhou, Guangdong, PR China; 2 Guangzhou Institute of Biomedicine and Health, Chinese Academy of Sciences, Guangzhou, Guangdong, PR China; 3 Institute of Biology, Guizhou Academy of Sciences, Guiyang, Guizhou, PR China; 4 Fanjingshan Forest Ecosystem Research Station, Guizhou Academy of Sciences, Jiangkou, Guizhou, PR China; Shanghai Jiao Tong University School of Medicine, China

## Abstract

Wogonin is a plant monoflavonoid which has been reported to inhibit cell growth and/or induce apoptosis in various tumors. The present study examined the apoptosis-inducing activity and underlying mechanism of action of wogonin in A549 cells. The results showed that wogonin was a potent inhibitor of the viability of A549 cells. Apoptotic protein changes detected after exposure to wogonin included decreased XIAP and Mcl-1 expression, increased cleaved-PARP expression and increased release of AIF and cytotchrome C. Western blot analysis showed that the activity of c-Myc/Skp2 and HDAC1/HDAC2 pathways, which play important roles in tumor progress, was decreased. Quantitative PCR identified increased levels of c-Myc mRNA and decreased levels of its protein. Protein levels of Fbw7α, GSK3β and Thr58-Myc, which are involved in c-Myc ubiquitin-dependent degradation, were also analyzed. After exposure to wogonin, Fbw7α and GSK3β expression decreased and Thr58-Myc expression increased. However, MG132 was unable to prevent c-Myc degradation. The present results suggest that wogonin has multiple anti-cancer effects associated with degradation of c-Myc, SKP2, HDAC1 and HDAC2. Its ability to induce apoptosis independently of Fbw7α suggests a possible use in drug-resistance cancer related to Fbw7 deficiency. Further studies are needed to determine which pathways are related to c-Myc and Fbw7α reversal and whether Thr58 phosphorylation of c-Myc is dependent on GSK3β.

## Introduction

Despite of the large number of clinical trials aimed at improving patient survival, lung cancer remains a leading cause of cancer-related mortality worldwide in both men and women. Approximately 85% of all lung cancer cases are categorized as non-small cell lung cancer (NSCLC), which is typically diagnosed at advanced stages [1]. Lung adenocarcinoma, the predominant histological subtype of NSCLC, accounts for 20 to 30% of primary lung cancer cases among subjects under 45 years of age, regardless of smoking history [2]. Most cases of NSCLC are unsuitable for surgery and chemotherapy remains the cornerstone of treatment for advanced disease.

Histone deacetylases (HDACs) are enzymes that remove histone acetylation products. This process compacts the structure of chromatin and represses transcription [3]. HDACs act on various nonhistone protein substrates which play a role in the regulation of gene expression, cell proliferation, cell migration, cell death and angiogenesis [4]. Data from preclinical studies have demonstrated that naturally occurring and synthetic histone deacetylase inhibitors have potent anticancer activity.

HDAC1 and HDAC2 belong to the Class I histone deacetylase (HDAC) family. In vivo, these enzymes form complexes with Sin3, NuRD and Co-REST [5]. HDAC1 and HDAC2 also bind directly to DNA binding proteins such as YY1, Rb binding protein-1 and Sp1 [5].

c-Myc is a transcription factor that is responsible for regulating an array of genes involved in cellular proliferation, growth, apoptosis and differentiation [6]. Deregulated c-Myc expression is observed in roughly 70% of all human tumors [10]. c-Myc expression is regulated by gene transcription, and is dependent on mRNA stability and posttranslational control of protein stability [7]–[9].

Posttranslational regulation of c-Myc can be mediated by Skp2 (S-phase kinase-associated protein 2) and Fbw7(F-box and WD repeat domain-containing 7) [11]. Skp2 and Fbw7 are two different recognition subunits of the SCF-type E3 ligase (SCF, Skp1/Cullin/F-box protein complexes) that recognize specific substrates for proteasomal degradation. Regulation of c-Myc involves phosphorylation of c-Myc at Thr58, resulting in Fbw7-mediated proteasomal degradation. Glycogen synthase kinase 3β(GSK3β) is the only kinase known to phosphorylate c-Myc at Thr58 [24].

Skp2 is a promising target for restricting cancer stem cell and cancer progression [12], it’s overexpression is frequently observed in human cancer and it may, therefore, act as an oncogene. In support of this hypothesis, Skp2 has been shown to recognize Cyclin-dependent kinase (Cdk) inhibitors and tumor suppressive proteins such as p27 Kip1 (Cyclin-dependent kinase inhibitor 1B), p57 Kip2 (Cyclin-dependent kinase inhibitor 1C), p130 (130 kDa retinoblastoma-associated protein) and Tob1 (transducer of ERBB-2 1), but not c-Myc [13]–[16]. Skp2-mediated formation of ubiquitin from c-Myc occurs independently of phosphorylation [25].

Fbw7 deficiency is thought to be involved in drug resistance in human cancers [22], [23]. It has been shown to be inactivated by mutation, deletion, or promoter hypermethylation in breast cancer [17], [18], colon cancer [19], [20], and leukemia [21]. Fbw7 is expressed as three different isoforms (designated α, β, and γ) respectively located in the α-nucleus, β-cytoplasm, and γ-nucleolus [26], [27]. As there are no antibodies for these three isoforms we used the best described and longest of the three isoforms Fbw7α, in our experiments with wogonin.

Wogonin (5, 7-dihydroxy-8-methoxyflavanon) is a naturally monoflavonoid extracted from *Scutellaria baicalensis* radix [28] that has been recognized as an anticancer drug candidate with potentially low toxicity [29]. The anticancer activity of wogonin has been reported various human cell lines including myeloma cell RPMI 8226 [30], hepatocellular carcinoma SK-HEP-1 [31] and SMMC-7721 [32], glioma cancer cells [33], nasopharyngeal carcinoma cells [34], human breast cancer cells [35], [36] and human cervical carcinoma HeLa cells [37]. Its activity is mediated by the induction of apoptosis and cell differentiation, and is regulated by various genes and proteins [38]–[41].

In this study, we evaluated the effects of wogonin on cell viability and apoptosis in the human lung adenocarcinoma epithelial cell line A549. We also assessed the regulation and function of c-Myc/Skp2/Fbw7α and HDAC1/HDAC2 pathways involved in its apoptotic effect.

## Materials and Methods

### Reagents and Antibodies

Wogonin was purchased from Guangzhou IDC (China) and MG132 (carbobenzoxy-Leu-Leu-leucinal) was obtained from (Beyotime, China). The following antibodies were used: Fbw7(Cdc4, H-300) and p-c-Myc (Thr 58) (Santa Cruz, USA); c-Myc, GSK3β, AIF (apoptosis inducing factor, Proteintech Group, Inc., USA); HDAC1, HDAC2, Skp2, Survivin, Bcl-2 (B-cell lymphoma 2), β-Actin (Boster, China); Mcl-1 (myeloid cell leukemia sequence 1), XIAP(X-linked inhibitor of apoptosis protein, Bioss, China); Cytochome c (KeyGEN, China); PARP (poly ADP-ribose polymerase, Sino Biological Inc., China).

### Cell Culture

The human pulmonary adenocarcinoma cell line A549 was obtained from the Cell Bank of the Animal Experiment Center, North School Region, Sun Yat-Sen University. The cells used in the experiments were maintained in our laboratory in RPMI 1640 medium with 10% fetal bovine serum (Sijiqing, China) at 37°C and 5% CO_2_.

### Methylthiazolyldiphenyl-tetrazolium Bromide (MTT) Cell Vaibility Assay

Cells harvested with trypsin were seeded into 96-well plates at a density of 1×10^4^ per well. After overnight incubation, the culture medium was removed and the cells were incubated with different concentrations of wogonin. After exposure to wogonin for 24, 48 or 72 h the cells were incubated with MTT at 37°C for an additional 4 h. This allowed mitochondrial dehydrogenase to convert MTT into insoluble formazan crystals. The culture medium was then discarded, and 100 µL of dimethylsulfoxide (DMSO) was added to each well to dissolve the formazan crystals. The absorption of solubilized formazan was measured at 490 nm using a EL340 microplate reader (Bio-Tek. Instruments, Winooske, VT).

### Nuclear Staining

A549 cells were stained with a DAPI (4′, 6-diamidino-2-phenylindole) staining kit (KeyGEN, China). After exposure to graded concentrations of wogonin for 48 h, the cells were washed and incubated with a DAPI working solution (1–2 µg/mL) for 15 min at 37°C. The cells were then rinsed with methanol and Buffer A (60% glycerol in 10 mM phosphate-buffered saline (PBS, pH7.6)) was added to the suspension. A549 cells were viewed using an Eclipse Ti Nikon microscope (Nikon, Japan).

### Mitochondrial Membrane Potential

The mitochondrial membrane potential (Δψm) of A549 cells was measured usimg a fluorescent, lipophilic, cationic probe, JC-1 (Beyotime, China), according to the manufacturer’s directions. Briefly, cells exposed to wogonin were incubated with 1X JC-1 staining solution for 20 min at 37°C. They were then rinsed twice with JC-1 staining buffer and images were taken with an Eclipse Ti Nikon microscope (Nikon, Japan).

### Apoptosis Assay

Cells were labeled with FITC-labeled Annexin V and propidium iodide (PI) using an Annexin V-FITC apoptosis detection kit (KeyGEN BioTECH, China), according to the manufacturer’s instructions. Briefly, after 48 h exposure to different concentrations of wogonin, the cells were washed with cold PBS and resuspended in 1X binding buffer. Aliquots of 10^5^ cells were mixed with 5 µL of Annexin V-FITC and 5 µL of PI for 15 min at room temperature in the dark. Fluorescence (530 nm) was detected by flow cytometry (FACS Aria, BD Biosciences, USA) within 1 h.

### Real-time PCR Analysis

Quantitative RT-PCR was undertaken using a SYBR Green reporter. A549 cells exposed to wogonin were washed with PBS and total RNA was purified by using RNAiso Plus (TAKARA, Japan). The resultant RNA was first reverse transcribed into cDNA using a PrimeScript® RT Master Mix kit (TAKARA, Japan). Gene-specific primers were combined with SYBR® Premix Ex Taq™ (TAKARA, Japan) and amplified using an ABI 7500 real-time PCR machine (Applied Biosystems, USA). All qPCR reactions were performed independently on five samples. The relative mRNA expression was calculated using the 2^−ΔΔCt^ method. The primer sequences used are listed in Table 1.

**Table 1:** Primer sequences for real-time PCRs.

Gene		Primer sequences (5′→3′)
GAPDH	Forward	GAAATCCCATCACCATCTTCCAGG
	Reverse	GAGCCCCAGCCTTCTCCATG
HDAC1	Forward	TAAATTCTTGCGCTCCATCC
	Reverse	AACAGGCCATCGAATACTGG
HDAC2	Forward	CGTGTAATGACGGTATCATTCC
	Reverse	ACCAGATAATGAGTCTGCACC
c-Myc	Forward	AGCGACTCTGAGGAGGAACAAG
	Reverse	GTGGCACCTCTTGAGGACCA
Skp2	Forward	TGGGAATCTTTTCCTGTCTG
	Reverse	GAACACTGAGACAGTATGCC
Fbw7α	Forward	AGTAGTATTGTGGACCTGCCCGTT
	Reverse	GACCTCAGAACCATGGTCCAACTT
GSK3β	Forward	GGCAGCATGAAAGTTAGCAGA
	Reverse	GGCGACCAGTTCTCCTGAATC

### Western Blot Analysis

Cells washed with PBS were lysed with RIPA (50 mM Tris (pH 7.4), 150 mM NaCl, 1% NP-40, 0.5% sodium deoxycholate, 0.1% SDS, sodium orthovanadate, sodium fluoride, EDTA and leupeptin, (Beyotime, China) supplemented with protease inhibitor PMSF (Beyotime, China). Cytoplasmic proteins were extracted using a Nuclear and Cytoplasmic Protein Extraction Kit (KeyGEN BioTECH, China).

The soluble protein concentration was determined with a BCA protein assay kit (Beyotime, China). The cell lysates were boiled for 5 min in loading buffer and separated on an SDS-PAGE gel. After electrophoresis, the proteins were transferred to a PVDF membrane (Millipore, USA). The membranes were blocked with nonfat milk, probed with various primary antibodies and HRP-conjugated secondary antibodies, and visualized with enhanced chemiluminescence (ECL) detection reagents (Beyotime, China).

### Statistical Analysis

Statistical analyses was undertaken using SPSS version 17.0 software. Results are expressed as means and standard errors (Mean ± SEM) from at least three independent experiments. One-way analysis of variance (ANOVA) was used to determine statistical significance between groups. Values of P<0.05 were considered statistically significant.

## Results

### Wogonin Inhibits Cell Viability and Induces Cell Apoptosis

**Figure 1:**
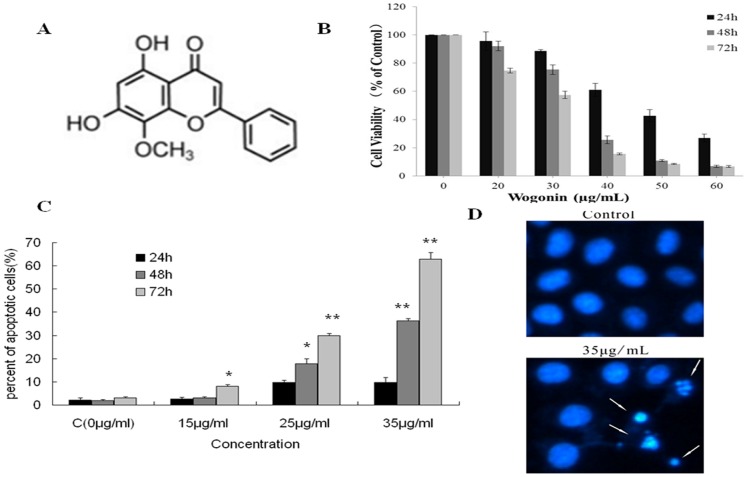
Chemical structure of wogonin and wogonin exerted potent anti- A549 activity *in vitro*. (A) Chemical structure of wogonin (C_16_H_12_O_5_, MW: 284.27). (B) Cell viability was analyzed using the MTT assay. Cells were incubated with wogonin for 24 h, 48 h or 72 h. The bars represent the mean values ± SEM (n = 3). (C) Apoptosis assessed by Annexin V/PI staining in A549 cells. Cells were incubated with wogonin at concentrations of 0, 15, 25, 35 µg/mL, for 24 h, 48 h and 72 h. (D) Nuclei stained by DAPI and observed by fluorescence microscope. Apoptotic cells are indicated by arrows. *P<0.05 vs control group, **P<0.01 vs control group.

Cell viability was assessed using an MTT assay in association with DAPI staining and flow cytometric analysis after exposure to different concentrations of wogonin. MTT analysis indicated that wogonin inhibited cell viability in a dose-dependent and time-dependent manner (Fig. 1B). This was confirmed by results of flow cytometric analysis using AnnexinV-PI (Fig. 1C).

Compared with the control group, the rate of both early and late stage apoptosis increased after exposure to all concentrations of wogonin. The total apoptosis rate exceeded 50% in the 35 µg/mL group.

DAPI staining (Fig. 1D) identified condensed and cleaved nuclei in cells exposed to 35 µg/mL wogonin, while only clear nuclei with pale blue staining were observed in the control group.

**Figure 2:**
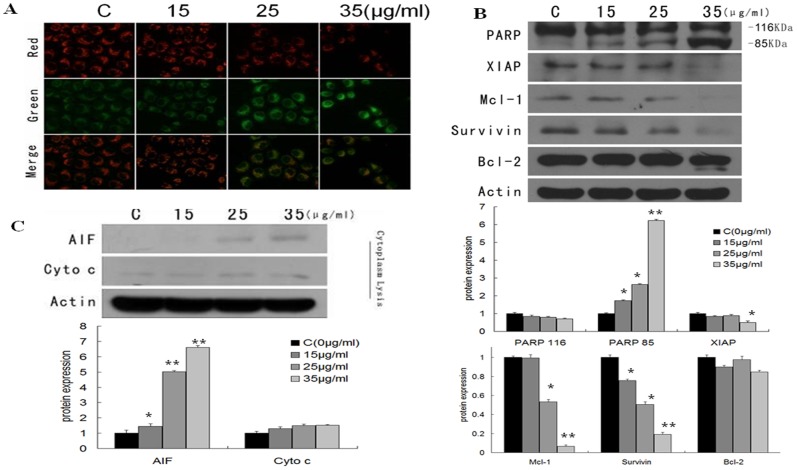
Effects of wogonin on mitochondrial membrane potential and apoptotic proteins in A549. (A) Analysis of the mitochondrial membrane potential (ΔΨm) using JC-1 staining after exposure to wogonin for 48 h. A fluorescence microscope was used to visualize the results. Mitochondrial depolarization was indicated by an increase in green fluorescence and a decrease in red fluorescence intensity. (B) Protein levels of Bcl-2, Mcl-1, XIAP, survivin and PARP assayed by western blot. (C) Cytoplasmic proteins were extracted for western blot analysis of released Cytochrome c and AIF. In these experiments, cells were exposed to wogonin 0, 15, 25, 35 µg/mL for 48 h. *P<0.05 vs control group, **P<0.01 vs control group.

As shown in Fig. 2A, Wogonin was associated with a dose-dependent decrease in mitochondria potential (Δψm) which resulted in decreased red fluorescence (JC-1 polymer) and increased of green fluorescence (JC-1 monomer). This may have been related to down-regulation of Mcl-1 as there was no obvious decrease in Bcl-2 levels. Wogonin also promoted the release of AIF and cytochrome C into the cytoplasm providing further evidence of mitochondrial damage (Fig. 2C). Down-regulation of XIAP, survivin, and of cleaved fragments from PARP indicated that the process of apoptosis continued after mitochondria damage (Fig. 2B).

### Wogonin Down-regulates HDAC1 and HDAC2 at Both mRNA and Protein Levels

**Figure 3:**
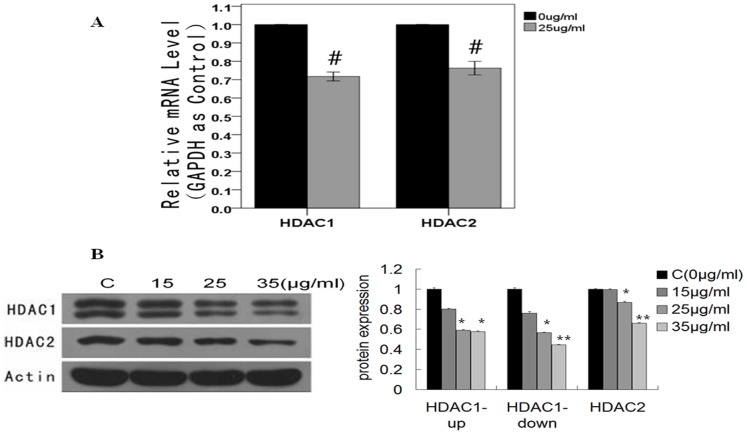
Effects of wogonin on HDAC1 and HDAC2 in A549. (A) Relative mRNA levels of HDAC1 and HDAC2 were detected using real-time PCR with GAPDH as an internal control. Results are expressed as the mean ± SEM of five independent experiments. ^#^ *P*<0.01 vs control group. (B) Protein levels of HDAC1 and HDAC2 assayed by western blot. In these experiments, cells were exposed to wogonin 0, 15, 25 and 35 µg/mL for 48 h. *P<0.05 vs control group, **P<0.01 vs control group.

Protein levels of HDAC1 and HDAC2 were down-regulated in a dose-dependent manner after exposure to different concentrations of wogonin for 48 h (Fig. 3B). This result is consistent with the down-regulation of mRNA detected by qPCR showing that HDAC1 was decreased by 0.69-fold and HDAC2 by 0.73-fold, (uncertainties related to fold-changes were <2) (Fig. 3A). These changes may result in a proportional increase in histone acetylation and promote the expression of tumor suppressive proteins.

### Wogonin Down-regulates c-Myc and Skp2 at the Protein Level, and Increases the mRNA Level of c-Myc

**Figure 4:**
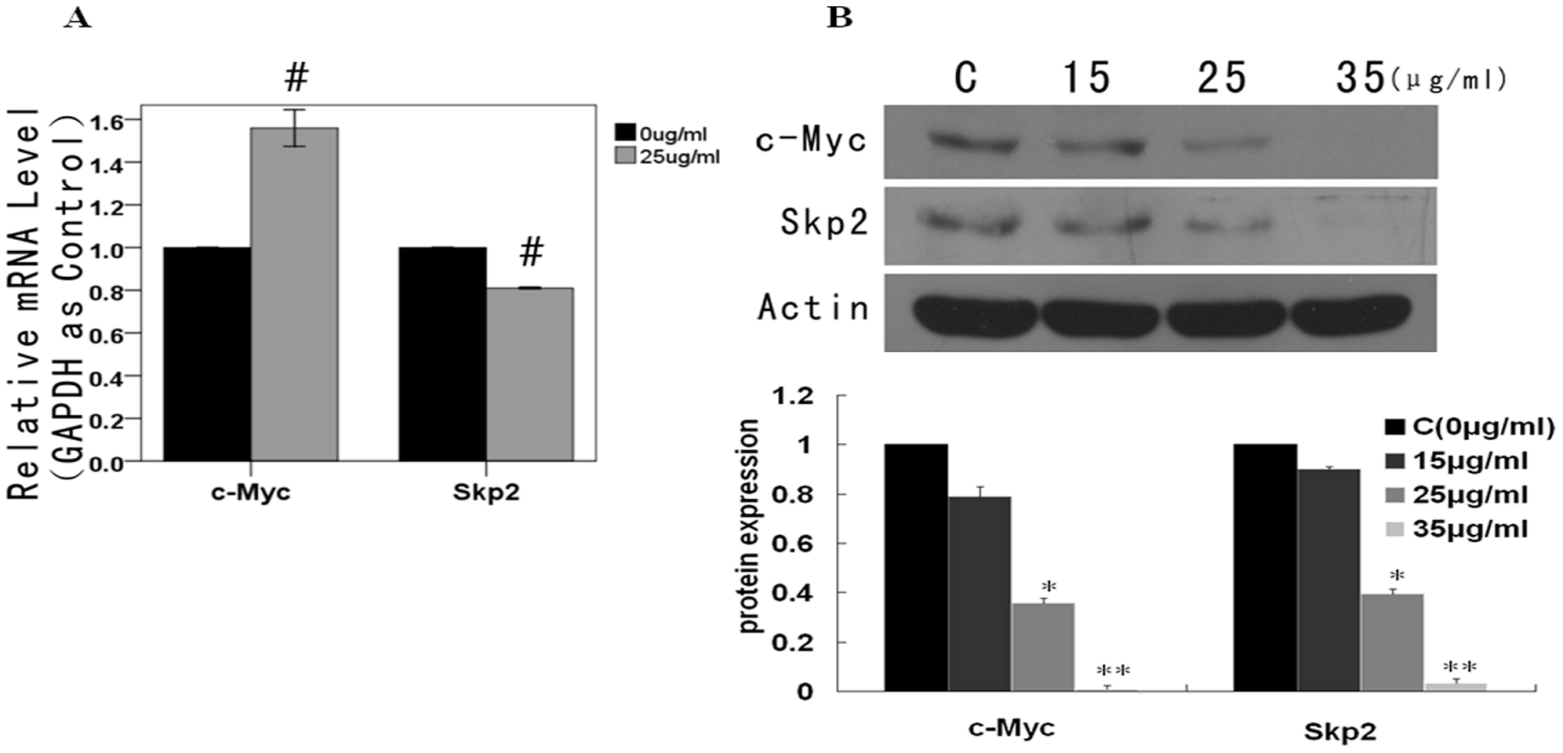
Effects of wogonin on c-Myc and Skp2 in A549. (A) Relative mRNA levels of c-Myc and Skp2 were detected using real-time PCR with GAPDH as an internal control. Results are expressed as the mean ± SEM of five independent experiments. ^#^ *P*<0.01 vs control group. (B) Protein levels of c-Myc and Skp2 assayed by western blot. In these experiments, cells were exposed to wogonin 0, 15, 25 and 35 µg/mL for 48 h. *P<0.05 vs control group, **P<0.01 vs control group.

Both c-Myc and Skp2 were down-regulated at the protein level following exposure to wogonin (0, 15, 25, 35 µg/mL) for 48 h, (Fig. 4B). As shown in Fig. 4A, the mRNA level of Skp2 decreased 0.81-fold, whereas the mRNA level of c-Myc increased approximately 1.6-fold (uncertainties related to fold-changes both <2). These findings indicate that a proteasomal degradation pathway may be involved in the regulation of c-Myc and Skp2. However, as wogonin resulted in decreased protein expression in Skp2, further experiments focused on the proteasome recognition subunit, Fbw7α, which targets c-Myc.

### Wogonin Decreased Fbw7α and GSK3β, and Increased Thr58-Myc at the Protein Level

**Figure 5:**
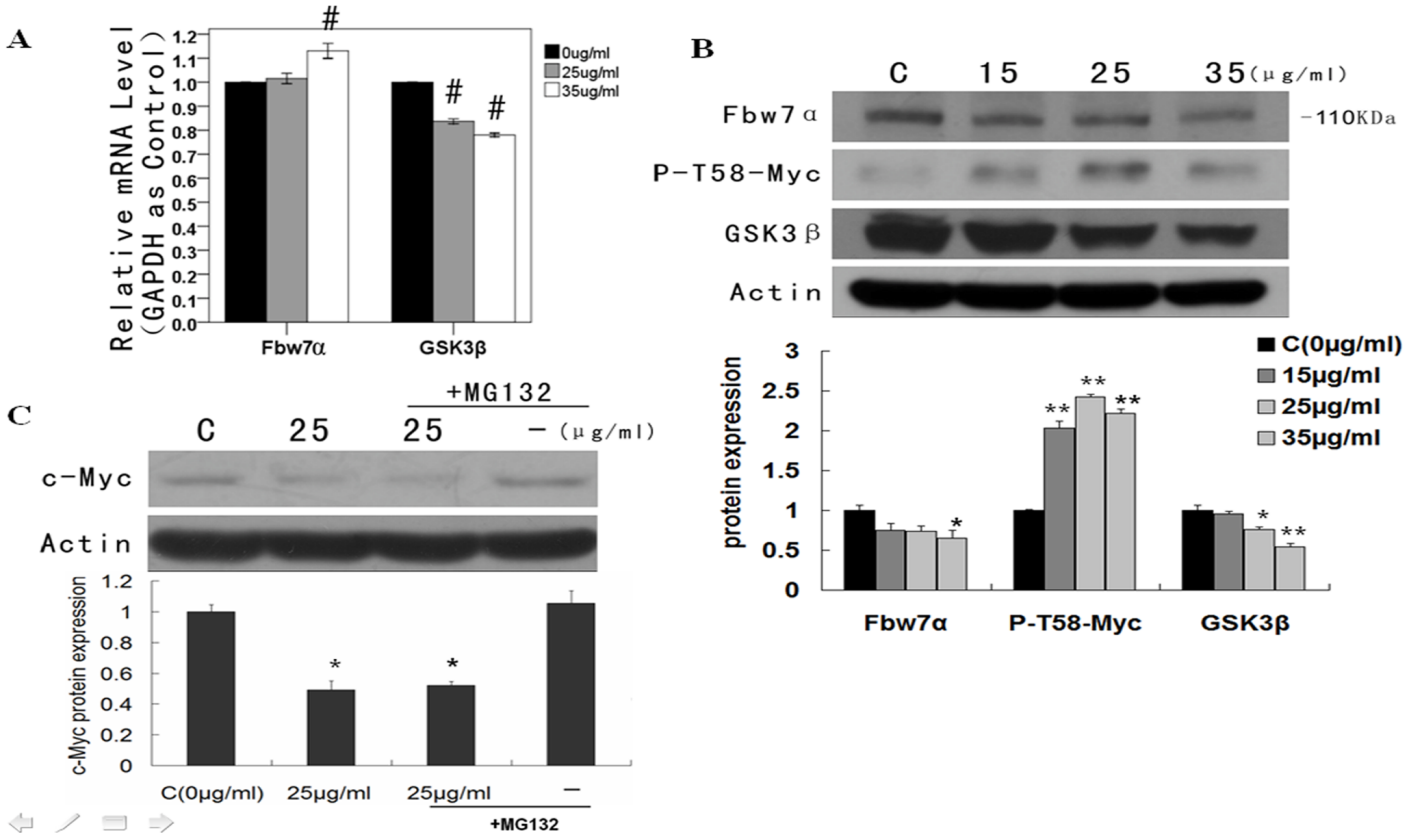
Effects of wogonin on Fbw7α and GSK3β in A549. (A) Relative mRNA levels of Fbw7α and GSK3β were detected using real-time PCR with GAPDH as an internal control. Results are expressed as the mean ± SEM of five independent experiments. ^#^ *P*<0.01 vs control group. (B) Protein levels of Fbw7α, Thr58-Myc and GSK3β assayed by western blot. (C) Protein levels of c-Myc assayed by western blot. In these experiments, 1 µM MG132 was added incubated with or without 25 µg/mL wogonin for 48 h. *P<0.05 vs control group, **P<0.01 vs control group.

Protein levels of Fbw7α decreased following exposure to wogonin, (Fig. 5B), but there was no corresponding decrease in mRNA expression (Fig. 5A). Thr58 phophorylation of c-Myc increased (Fig. 5B). Thr58 phophorylation of c-Myc is a required component of its degradation and is mediated by GSK3β. However, GSK3β expression decreased at both the mRNA (0.78-fold at 35 µg/mL) (Fig. 5A) and protein level (Fig. 5B). These findings suggest that phophorylation of c-Myc at Thr58 may occur independently of GSK3β. However, this requires further study.

Experiments were conducted with the proteasome inhibitor MG132 to further understand the proteasomal degradation pathway involved in the regulation of c-Myc. The results in Fig. 5C show that MG132 was unable to reverse c-Myc degradation induced by 25 µg/mL wogonin. Further research is therefore needed to define the exact pathway involved in c-Myc degradation.

## Discussion

Wogonin is a naturally occurring monoflavonoid extracted from *Scutellaria baicalensis* radix [28]. It has been reported to have antineoplastic activity in various types of cancer by the induction of apoptosis and cell differentiation [30]–[37], and to be regulated by various genes and proteins [38]–[41]. It is known that the c-Myc/Skp2/Fbw7α and HDAC1/HDAC2 pathways, are associated with tumor progression. Here we investigate their role in the anticancer effects of wogonin in NSCLC A549 cells.

We first evaluated the anti-viability and apoptotic effects of wogonin using MTT and Annexin V-PI double staining assays. Our results indicated that wogonin caused dose-dependent and time-dependent inhibition of cell viability (IC_50_<35 µg/mL at 48 h). The early increase in apoptosis rate in response to wogonin occurred in parallel, with Annexin V-positive cells gradually becoming Annexin-V negative.

At the IC_50_ (35 µg/mL) cells showed evidence of marked apoptosis, consistent with the nuclear morphology changes seen with DAPI staining.

The expression of apoptosis related proteins, such as Bcl-2, Mcl-1, PARP, XIAP, Survivin, cytochrome c and AIF was evaluated to further identify the apoptotic effects of wogonin at the protein level. The results indicate that wogonin is able to influence mitochondrial membrane stability and decrease mitochondria membrane potential (Δψm). This was evidenced by JC-1 staining, decreased mcl-1 expression and the release of cytochrome C and AIF into the cytoplasm. Cleaved PARP and decreased XIAP and survivin expression may also have contributed to the progression of apoptosis.

HDAC1 and HDAC2 are Class I HDACs that deacetylate histone and non-histone proteins [5]. They suppress gene expression, and modify tumor specific proteins involved in progression [4]. Our results show that the mRNA and protein levels of HDAC1 and HDAC2 were both decreased in the presence of wogonin, indicating that acetylated histone protein may promote expression of tumor suppressive proteins and thereby inhibit tumor progression.

An inter-relationship exists between c-Myc and Skp2 such that c-Myc promotes Skp2 expression, and Skp2 targets c-Myc for ubiquitin-dependent degradation [11], [12], [24], [25]. In our experiments, wogonin down-regulated c-Myc and Skp2 at the protein level, but the mRNA expression of c-Myc increased 1.6-fold. These findings suggest that the proteasomal degradation pathway may be related to the reversal of c-Myc. However, since Skp2 expression decreased, we focused our further experiments on Fbw7α, another proteasome recognition subunit that targets c-Myc.

Thr58 phophorylation of c-Myc is required for Fbw7α mediated c-Myc degradation [24]. As Thr58 is phophorylated by GSK3β, we evaluated expression levels of Fbw7α, Thr58 c-Myc and GSK3β. In these experiments Fbw7α expression decreased at the protein level but not at the mRNA level. Thr58 phophorylation of c-Myc increased to some degree and GSK3β expression at both the mRNA and protein level decreased. These results highlight lack of conformity between Fbw7α mRNA and protein levels, the decreased expression of GSK3β, and the increased phophorylation of c-Myc at Thr58.

A previous study demonstrated that phosphorylation of Thr58 in A549 cells occurred independently of GSK3β [42]. However, GSK3β is the only known kinase that phosphorylates c-Myc at Thr58. In our studies the proteasome inhibitor MG132 was unable to prevent the degradation of c-Myc suggesting that decreased Fbw7α and Skp2 might be involved in this process. However, the exact mechanism involved requires further research.

Taken together our findings suggest that the ability of wogonin to influence different biochemical pathways may explain its activity against a variety of different cancers. Our data may also in part explain why some gene deficient cells (e.g. those with Fbw7α deficiency) are resistant to wogonin.
